# Advancements in Active-Pixel-Type CMOS Image Sensor Design Techniques and Architectures for Wide Dynamic Range

**DOI:** 10.3390/s26020489

**Published:** 2026-01-12

**Authors:** Sangwoong Sim, Jaehoon Jun

**Affiliations:** Department of Electrical and Computer Engineering and Program in Semiconductor Convergence, Inha University, Incheon 22212, Republic of Korea; aioseu79@inha.edu

**Keywords:** CMOS image sensor (CIS), correlated multiple sampling (CMS), dual conversion gain (DCG), dual photodiode (PD), dynamic range (DR), lateral overflow integration capacitor (LOFIC), linear response, linear–logarithmic (Lin-Log) response, logarithmic response, multiple exposure

## Abstract

Advances in CMOS image sensors (CISs) have led to utilization in various industrial fields, including machine vision, medical, surveillance, the automotive industry, and the Internet of Things (IoT). One critical metric for CISs is the dynamic range (DR), which indicates the range of light intensity that can clearly capture images. As the technology evolves, wide dynamic range (WDR) becomes increasingly required for more diverse applications. To further advance these industries, this paper presents the active-pixel-type CIS design techniques and architectures developed to achieve WDR. These include the following: the basic concepts of the active pixel sensor, readout mechanism, and DR of the CIS; multiple exposure and dual conversion gain (DCG) schemes that are conventionally used to address a trade-off in the CIS; lateral overflow integration capacitor (LOFIC) and dual photodiode (PD) architectures that can improve the DR by utilizing trade-offs in the DR and exposure mechanism; CISs with logarithmic and linear–logarithmic (Lin-Log) responses to enable non-linear characteristics; and techniques that can be employed for higher sensitivity in dark conditions. This comprehensive study of various techniques and architectures can also be utilized for cutting-edge tech advances and future research, including neuromorphic array architecture.

## 1. Introduction

In the late 20th century, CMOS image sensors (CISs) began to be developed in earnest due to their significant potential, such as low system-level cost, low power consumption, and high-speed readout relative to charge-coupled devices (CCDs). Despite these potential benefits, CISs were not widely commercialized in the industry due to their lower image quality and higher noise compared to CCDs. However, modern CISs have advanced to achieve higher resolution, lower power consumption, and faster imaging than CCDs, while inherently providing random-access readout and effective suppression of blooming and smearing [1]. As a result, they have been widely utilized in a variety of industries, including machine vision, surveillance, the automotive industry, and the Internet of Things (IoT). Recently, advanced applications of artificial intelligence (AI), such as intelligent vision sensors [2,3,4] and image recognition based on memristors [5,6], have also attracted considerable attention. In these industries, one critical performance metric for CISs is the dynamic range (DR), indicating the range of light intensity over which images can be clearly captured. However, conventional CISs based on 3-transistor (3T) and 4-transistor (4T) types provide a DR of around 60 dB, which is insufficient for capturing images in extremely bright and dark conditions. As diverse industrial fields require extensive light intensity, improving the DR of CISs has become imperative.

The DR is defined as the ratio of the highest detectable illumination level to the lowest detectable illumination level, which varies depending on the pixel configuration and the exposure time. At the pixel level, extending full-well capacity (FWC) allows more photons to be accumulated under bright conditions, while increasing conversion gain (CG) improves sensitivity under dark conditions. Similarly, in terms of the exposure time, a short exposure prevents saturation in bright areas, whereas a long exposure enables the capture of dark objects. However, the DR of conventional single-exposure CISs is constrained by two interrelated trade-offs: one between the FWC and CG and another between the short and long exposure times. To address these limitations, this paper introduces techniques and architectures that can be adaptively utilized in different fields requiring the wide dynamic range (WDR). First, a brief overview of techniques and architectures with a linear response is presented. The multiple exposure technique [7,8,9,10,11,12,13,14,15,16,17] synthesizes images by utilizing different integration times within a single frame. Through digital processing of composite images, the DR is improved without modifying pixel configurations. The dual conversion gain (DCG) architecture [18,19,20,21,22,23,24,25,26,27,28,29,30,31] takes two readouts, first with high conversion gain (HCG) for high sensitivity, and second with low conversion gain (LCG) for large FWC. This approach solves the trade-off with the CG and FWC, resulting in high DR. The lateral overflow integration capacitor (LOFIC) structure [32,33,34,35,36,37,38,39,40,41,42,43,44,45,46,47] accumulates overflowed charges when the photodiode (PD) saturates under extremely high illumination. It has high-linear FWC and high sensitivity while extending the DR by reading out overflowed charges. The dual PD architecture [48,49,50,51,52,53,54,55,56] utilizes two PDs of different sizes. It expands the DR based on the sensitivity ratio of a large photodiode (LPD) and a small photodiode (SPD). The dual PD scheme can be simple to incorporate with other approaches, which are utilized to further extend the DR.

The discussion has shifted to the non-linear response. CISs with a logarithmic response [57,58,59,60,61,62,63,64,65,66] read out the voltage formed by the current flowing through the PD. Unlike CISs with a linear response, which convert charge to voltage linearly, these use a weak inversion in which the current forms the gate-source voltage logarithmically. This results in higher DR compared to the linear response without increased FWC. However, under low illumination, the signal-to-noise ratio (SNR) of the logarithmic response is too low, making it difficult to capture clear images. To address this issue, CISs with a linear–logarithmic (Lin-Log) response [67,68,69,70,71,72,73,74,75,76,77,78] were devised that utilize a linear response in dark conditions and a logarithmic response in bright conditions.

Finally, the focus is on schemes to improve the DR by reducing noise. The SNR of CISs in low-light intensity is limited by the fixed pattern noise (FPN), reset noise, and readout noise (RN), typically caused by the analog readout operation. The FPN and reset noise can be reduced by the correlated double sampling (CDS) [79,80,81]. The RN can be decreased to a sub-electron level in the analog domain primarily through two methods: by increasing the CG or by implementing high gain at the analog front-end. The CG is determined by the parasitic capacitor of the floating diffusion (FD). Thus, by reducing the parasitic capacitors, which are caused by the transfer gate (TG) and reset gate (RG), it is possible to achieve significantly larger CG [82,83,84,85,86,87]. The high gain at the analog front end can be achieved utilizing the dual-gain column amplifiers [88,89] and the source followers (SFs) as the differential common source (CS) amplifier [90,91]. In addition, the RN can also be reduced in the digital domain through correlated multiple sampling (CMS) [92,93,94,95,96,97,98,99,100]. CMS achieves low noise by repeatedly reading out the same pixel and processing the sampled data. These approaches result in high sensitivity, which expands the DR under low illumination. While a single-photon avalanche diode (SPAD) [101], a quanta imaging sensor (QIS) [102], and neuromorphic vision sensors [103] can be employed for extremely low light or event-driven imaging with asynchronous readout, this paper focuses on typical techniques and architectures for achieving WDR under both bright and dark conditions.

Accordingly, this paper delves into the techniques and architectures of active-pixel-type CIS for WDR. Section 2 introduces the conventional active pixel sensor (APS), the readout mechanism of rolling shutter and global shutter, and the details of the DR to provide a basic understanding. Section 3 discusses the multiple exposure and DCG schemes, while Section 4 focuses on the LOFIC and dual PD architectures. Section 5 covers the logarithmic and Lin-Log responses, followed by approaches to extend the DR under low illumination in Section 6. Finally, Section 7 concludes this paper.

## 2. CMOS Image Sensors

The conventional passive pixel sensor (PPS) scheme was proposed in 1967. It consists of a one-pass transistor between the PD and the column output line. Because of the use of only a single transistor and PD, PPSs achieve high fill factors and small pixel sizes. However, PPSs have problems with the RN and frame rate. To address these, the conventional APSs were developed [104,105,106].

### 2.1. Active Pixel Sensor

**Figure 1:**
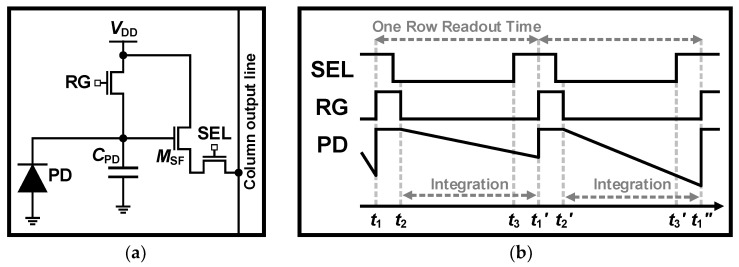
(**a**) Schematic and (**b**) timing diagram of conventional 3T APS.

Figure 1a presents a schematic of the conventional 3T APS, the most fundamental structure of APS. The 3T APS consists of one PD and three transistors: the RG, SF, and row selection switch (SEL). The RG removes residual electrons from the PD and functions like an electronic shutter, the SF functions as a buffer, and the SEL determines which row pixels are read out in the column output line. Figure 1b shows the operation of the 3T APS. At *t*_1_, the RG is turned on to remove residual charges from the previous readout, resetting the PD. After *t*_2_, the RG is turned off, allowing the PD to accumulate charges, at which point its voltage reduces linearly relative to light intensity. Before the fixed integration time ends, the SEL is turned on at *t*_3_, and the voltage of the PD is read out.

The readout operation in the APS introduces the FPN and reset noise, significantly affecting the quality of the image. In the 3T APS, the conversion of light to charge and charge to voltage occurs at the same node. This leads to the use of delta reset sampling (DRS) to reduce the FPN and 1/*f* noise [39,43,106]. DRS is a method that measures the output signals at two time points, before and after a pixel reset, and subtracts the difference between the two signals. Since two signals are uncorrelated, it can suppress the FPN and 1/*f* noise but cannot eliminate the reset noise. Therefore, the 3T APS is rarely used when precision is critical. Instead, the 4T APS that resolves this problem through the CDS can be employed.

**Figure 2:**
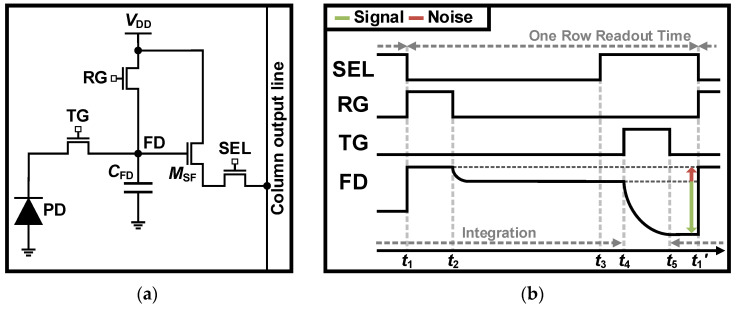
(**a**) Schematic and (**b**) timing diagram of conventional 4T APS.

**Figure 3:**
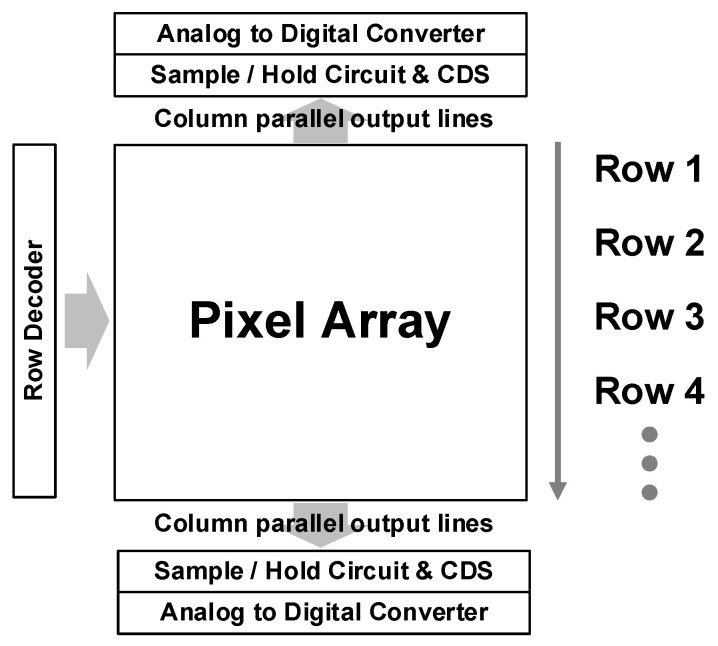
Chip architecture of CMOS image sensor for CDS.

Figure 2a illustrates the conventional 4T APS, which consists of the 3T APS with the TG. In the 4T APS, the FD capacitor (*C*_FD_) temporarily holds charges after the integration time, allowing the integration of the next frame while reading out the signal of the FD. Therefore, the 4T APS can achieve faster frame rates than the 3T APS. The timing diagram of the 4T APS is shown in Figure 2b. Initially, the integration starts during the readout of the previous frame; then, the RG is switched on to reset the FD for use as storage at *t*_1_. The RG is switched off at *t*_2_, resulting in the reset noise. During the integration time, the noise is read out at *t*_3_ with the SEL enabled, then sampled through the sample-and-hold circuit for the CDS in Figure 3. At *t*_4_, the TG is turned on to convert the integrated charges from the PD to the voltage of the FD. This voltage, which includes the signal and noise, is read out when this settles at *t*_5_. The charge on the PD is transferred across this point, enabling the integration for the next frame at *t*_1_’. In this process, the CDS circuit subtracts the two readout signals to cancel out the FPN, reset noise, and 1/*f* noise. Implementation of the CDS is possible in analog, digital, or a hybrid of both [79,80,81]. Additionally, the timing of the read operation can be optimized to ensure high frame rates with the CDS [107]. Consequently, the 4T APS improves the SNR and operating speed compared to the 3T APS by only adding the TG.

### 2.2. Readout Mechanism

**Figure 4:**
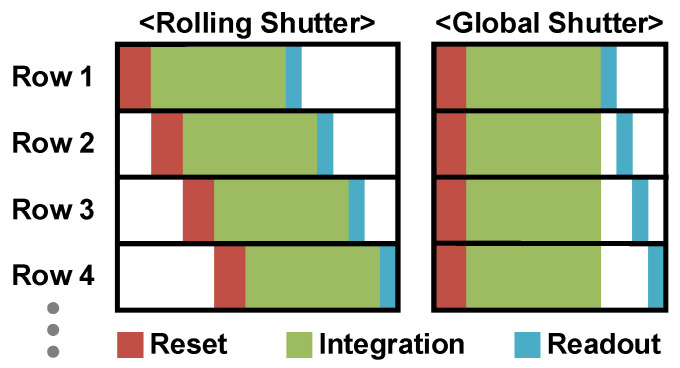
Readout timing of rolling shutter and global shutter.

CISs can be classified into two types based on their readout mechanism. One operates as the rolling shutter readout, which exposes and reads each row of pixels sequentially, as depicted in Figure 4. The rolling shutter offers higher resolution, lower manufacturing costs, and lower RN compared to the global shutter, resulting in higher image quality. However, the rolling shutter causes distortions when capturing fast-moving objects, known as motion artifacts. For this reason, the global shutter readout, another type of CIS, can be employed. The global shutter exposes all row pixels simultaneously and reads them out row by row. This mechanism eliminates distortion as all pixels capture the image at the same time, and it provides faster readout than the rolling shutter. To implement the global shutter, each pixel must have in-pixel storage to preserve its charges. Depending on the pros and cons of each, there are different approaches to having the in-pixel storage, such as the FD, MOS storage gate (SG), and pinned charge storage diode (SD) [108]. However, it is difficult to have high performance in terms of the DR, quantum efficiency (QE), and dark current with the global shutter. In addition, the global shutter requires costs related to resolution, area, and power. In this paper, the rolling shutter will be referred to as the default from this point forward.

### 2.3. Dynamic Range

**Figure 5:**
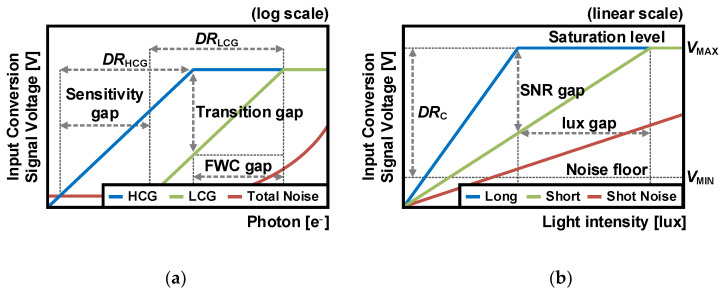
(**a**) Relationship between FWC and sensitivity with the variation in CG, and (**b**) relationship between SNR and light intensity with the variation in integration time.

The DR of the conventional single-exposure APS, *DR*_C_, is defined as the ratio of the highest detectable illumination level to the lowest detectable illumination level as follows:

*DR*_C_ = 20log [FWC/(*N*_FPN_ + *N*_reset_ + *N*_RN_ + *N*_shot_)] (1)

where FWC is the number of photons that the *C*_FD_ fills to its full capacity, and *N*_FPN_, *N*_reset_, *N*_RN_, and *N*_shot_ are defined as the FPN, reset noise, RN, and shot noise, respectively. As depicted in Figure 5a, an increase in parasitic capacitance leads to an expansion of the FWC, resulting in the LCG, which reduces sensitivity in low-light conditions. Conversely, a decrease in parasitic capacitance results in HCG, which increases sensitivity but reduces the FWC compared to the LCG. In other words, there is a trade-off between the FWC and CG, limiting the DR of the APS.

The *DR*_C_ can be expressed in terms of the signal voltage level as follows:

*DR_C_* = 20log (*V*_MAX_/*V*_MIN_) (2)

where *V*_MAX_ represents the saturation level in high-light conditions, and *V*_MIN_ represents the point where the noise and signal intersect in low-light conditions. As shown in Figure 5b, at the same light intensity, a longer integration time results in more charges being converted and a higher signal level. This means that a longer integration time has a higher SNR before the signal reaches the saturation level. In contrast, a short integration time can have more light intensity since relatively fewer charges are converted to a signal. This trade-off is important for the multiple exposure technique, which will be discussed in more detail in the next section.

The DR represents the range of light intensity that can be captured in the CIS. The upper limit of light intensity requires expanding the FWC or the short exposure, while the lower limit demands increasing the CG or the long exposure for high sensitivity. As previously mentioned, because the FWC and CG are inversely correlated, conventional single-exposure CISs are limited to a DR of 65–75 dB. However, it is insufficient for various applications used for extremely bright and dark environments. For this reason, various approaches have been devised to achieve WDR that performs well under both high and low illumination. The following two sections describe the general techniques and architectures used for expanding the DR.

## 3. Trade-Off Solutions for Wide Dynamic Range

First, Section 3 discusses the multiple exposure technique and DCG architecture to address the trade-offs described in Figure 5.

### 3.1. Multiple Exposure

An important feature of the 4T APS is the ability to control the integration time by the RG [7]. When shooting a sunlit window indoors, the long integration time results in saturated pixels in sunlit areas, while the short integration time is insufficient to capture dark objects. In this extreme condition, by synthesizing two images with different integration times, clear images can be captured in both bright and dark areas. Increasing the DR in this way is known as the multiple exposure technique. This utilizes short-exposure data for bright areas and long-exposure data for dark areas to digitally achieve WDR.

**Figure 6:**
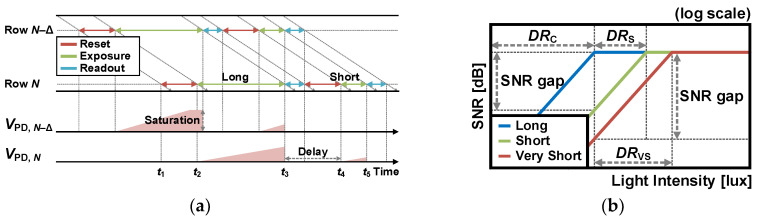
(**a**) Timing diagram with long and short exposures, and (**b**) relationship between DR and SNR with the variation in integration time.

The timing diagram of multiple exposure with long and short exposures [8,9] is shown in Figure 6a, where *V*_PD,*N*_ and *V*_PD,*N*–Δ_ represent the voltages converted by photons in rows *N* and *N*–Δ, respectively. In the readout operation, the pixels of row *N* are reset at *t*_1_, and a long exposure begins at *t*_2_. After integration is completed at *t*_3_, the *V*_PD,*N*_ is read out to the downside column-parallel signal chain, as shown in Figure 3. At the same time, the *V*_PD,*N*–Δ_ of a short exposure needs to be read out, so this is achieved with the upside column-parallel signal chain. After the delay, a short exposure of row *N* begins at *t*_4_, and the *V*_PD,*N*_ is read out at *t*_5_. Because there is a latency between the long exposure signal and the short exposure signal, to combine the data of different times, delay line memory (vertical shift register) is necessary for image synthesis [10,11]. To resolve this issue, in-pixel multiple-exposure synthesis can be used [12].

As depicted in Figure 5b, varying exposure times result in different saturated light densities. To achieve the same signal level with different exposures, the light intensity for a short exposure must be as large as the ratio of exposure times. In other words, this means that the DR of the multiple exposure technique, *DR*_M_, increases as the ratio of periods as follows:

*DR*_M_ = *DR*_C_ + 20log (*T*_long_/*T*_short_) (3)

where *T*_long_ is the longest exposure time, and *T*_short_ is the shortest exposure time. For example, an exposure ratio of 10 improves the DR by 20 dB. However, as the ratio increases, the SNR gap in the transition of different exposure signals becomes larger, as depicted in Figure 6b. In addition, in high-light conditions, the shot noise becomes dominant, reducing the SNR. These restrictions can be addressed by appropriately setting the integration time ratios of multiple exposures. This means that the multiple exposure technique can achieve WDR while maintaining high SNR in all DR regions, depending on the desired light intensity [13,14,15,16]. However, as multiple exposure requires additional integration times and synthesizes signals with delay, it reduces the frame rate and exacerbates motion artifacts, including missing parts of objects, edge color artifacts, and object distortion. To mitigate these limitations, the line interleave method [17], where two lines (rows) have different integration times, and the coded exposure method [109] can be employed.

In automobile applications, it is essential to be able to detect traffic lights and head–tail lights of cars (which can flicker several hundred times per second), as well as the environment of attention with high color reproducibility for all illumination conditions. When capturing traffic lights with multiple exposure, short integration is not able to detect light information if the light is off due to the light-emitting diode (LED) flicker. To address this problem, an approach was proposed for LED flicker mitigation (LFM), which operates by integrating single exposure into cycle repetitions [110,111]. In summary, the multiple exposure technique can have HCG for high sensitivity, and WDR can be achieved by adjusting the exposure times without the addition of transistors to the APS. In contrast, it requires faster readout circuitry for mitigating the motion artifact and on-chip line memory, resulting in increased die size and power consumption.

### 3.2. Dual Conversion Gain

CG in the 4T APS is determined by the *C*_FD_ due to the parasitic capacitors of the TG and the RG. However, reducing the *C*_FD_ achieves high sensitivity but lowers the FWC. To achieve WDR by resolving this trade-off, HCG and LCG can be achieved simultaneously by adding a gate with the capacitance of the *C*_DCG_, called the DCG architecture.

**Figure 7:**
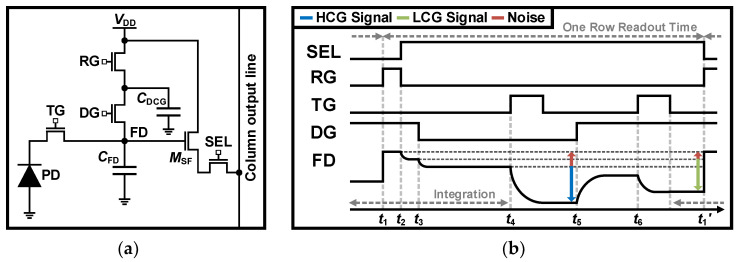
(**a**) Schematic and (**b**) timing diagram of DCG architecture.

The schematic and readout operation of DCG architecture is shown in Figure 7, where the DG is a gate between the *C*_FD_ and *C*_DCG_ that controls the HCG and LCG modes. First, the *C*_FD_ and *C*_DCG_ are reset at *t*_1_. After *t*_2_, the noise of the LCG mode is sampled. For high sensitivity, the DG is turned off into HCG mode at *t*_3_, and the noise of HCG is sampled. After integration time, the TG is turned on to transfer the charges from the PD to the FD at *t*_4_, and the conversion signal of HCG is read out. To achieve high-linear FWC, at *t*_5_, the DG is switched on to be in LCG mode. Then, the TG is turned on once more at *t*_6_ because the FWC of the FD is not sufficient to convert all charges in the PD at once under high illumination. After that, the conversion signal of LCG is read out. In this case, all signals can be processed by the CDS to remove noise.

The DR of the DCG, *DR*_D_, is determined by the *C*_DCG_ as follows:

*DR*_D_ = *DR*_C_ + 20log [(*C*_DCG_ + *C*_FD_)/*C*_FD_]. (4)

This means that as the *C*_DCG_ increases, the difference in CGs becomes larger, extending the *DR*_D_. This DCG architecture achieves a WDR of about 90 dB [18,19,20] by adding only one transistor in the 4T APS while maintaining the high fill factor and QE [21]. To have more large-linear FWC, i.e., to further increase the DR, an additional capacitor can be utilized. However, this requires a large area and restricts the pixel pitch, fill factor, and QE. To address these problems, stacking structures can be employed [22,25]. As illustrated in Figure 5a, larger CG differences increase the DR. However, at the transition point from HCG to LCG, the signal is reduced by *C*_FD_/(*C*_FD_ + *C*_DCG_), which increases the SNR gap. This problem also affects the LOFIC, which will be discussed in Section 4.

**Figure 8:**
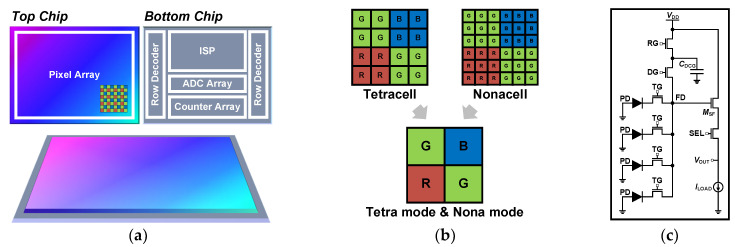
(**a**) Simplified architecture of 3-D-stacked CIS. (**b**) Transitions of tetracell and nonacell to tetra and nona modes, and (**c**) schematic of tetracell with DCG.

CISs with hundreds of megapixels are being used in state-of-the-art mobile applications. For high-resolution systems, three-dimensional (3-D) wafer-level stacked CISs have been developed [112,113]. As depicted in Figure 8a, a 3-D-stacked CIS architecture commonly consists of a top chip composed of the pixel array and a bottom chip integrating the analog front-end and digital logic. By independently optimizing the top and bottom chips, this architecture can achieve submicron pixel pitch with reduced dark current and enhanced QE. Furthermore, the bottom chip can employ a more advanced process node than the top chip, enabling reduced power consumption and increased speed. However, reducing the pixel pitch limits the FWC of the PD, which consequently restricts the DR. To mitigate this problem, the tetracell or nonacell design was combined with the DCG structure [23,24,25]. The tetracell and nonacell structures consist of four and nine PDs connected to a single FD, respectively, as shown in Figure 8b. In tetra and nona modes, where all connected PDs function as a single pixel, these schemes enhance sensitivity in low-light intensity while providing higher frame rates. Moreover, as shown in Figure 8c, the DCG structure can be combined to increase linear FWC, thereby achieving WDR. However, achieving sufficient FWC remains challenging as the pixel pitch becomes smaller. For this reason, a method of sharing the *C*_DCG_ between vertical pixels [25,26] and a structure with triple CGs [26] were devised.

The DCG scheme takes readouts in a single exposure, so this is less affected by motion artifacts and LED flicker than the multiple exposure technique. However, the *DR*_D_ can have values near 90 dB. To extend the DR, a combination of the DCG architecture and the multiple exposure technique was proposed to have 120 dB [27]. In addition, because portable devices require low power dissipation, a structure that utilizes decision feedback was proposed [28]. The DCG method needs two readouts, so a technique that performs only one readout by determining the HCG or LCG according to the threshold value through pre-readout was also proposed for higher frame rates [29,30]. Furthermore, as the LCG signal is intended for high illumination, a scheme with digital offset from the transition point was devised [31].

## 4. Utilizing Trade-Offs in Dynamic Range and Exposure Mechanism

In this section, the LOFIC and the dual PD architectures are described. These schemes can achieve WDR based on the principles covered in the previous section.

### 4.1. Lateral Overflow Integration Capacitor

The LOFIC architecture originated from the lateral overflow drain (LOD) structure in CCDs [114], which was developed to prevent blooming by discharging excess charges through an overflow drain. In particular, an LOD based on a junction field effect transistor evolved into the first LOFIC by adding a gate that controls the surface potential [115]. However, minimizing the LOD area was necessary to maintain high QE as the pixel pitch decreased. With the advancement of CMOS technology, this limitation was overcome through the transition from the LOD to the LOFIC [116].

**Figure 9:**
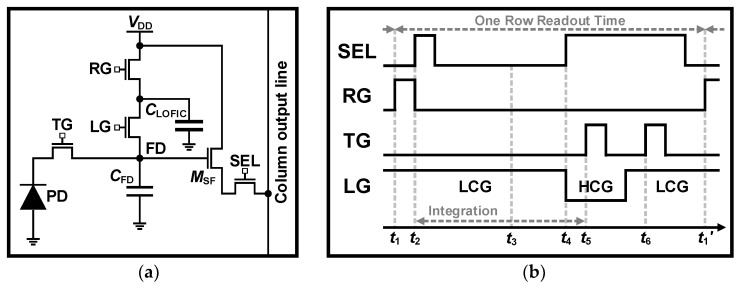
(**a**) Schematic and (**b**) timing diagram of LOFIC architecture.

**Figure 10:**
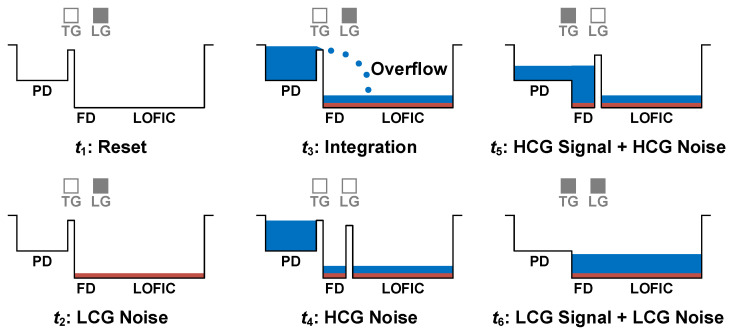
Potential well diagrams of LOFIC architecture operating under high illumination.

The key principle of the LOFIC is to extend the DR by storing overflowed charges from the PD in a capacitor with large-linear FWC. This allows the LOFIC architectures to have high sensitivity and much higher FWC over the DCG structure, achieving DRs of 100 dB [32,33,34,35,36]. As illustrated in Figure 9, the readout operation is similar to the DCG structure, so the potential well diagram shows the differences to be seen, as depicted in Figure 10. As shown in Figure 9a, except for the *C*_LOFIC_, the concept of the LOFIC structure is similar to the DCG structure, where the LG is a gate to control the DCG mode like the DG. Figure 9b shows the timing diagram corresponding to Figure 10. The *C*_FD_ and *C*_LOFIC_ are reset at *t*_1_. When the RG is turned off at *t*_2_, the integration is started, and the noise of LCG is sampled. If it is in bright conditions, the overflow occurs at *t*_3_, i.e., overflowed charges accumulate in the *C*_FD_ and *C*_LOFIC_. At *t*_4_, the integration is over, and the LG is switched off so that the accumulated charge is divided according to the capacity of the *C*_FD_ and *C*_LOFIC_. Then, the noise of HCG, which partly contains the overflow signal, is sampled. When the TG is turned on at *t*_5_, the charge of PD is transferred to the FD, and the signal of HCG is then read out after the TG is turned off. Finally, to read out all the charges, the TG and LG are activated at *t*_6_. After the TG is switched off, the signal of LCG is read out. Since this requires two readouts—both the DCG and LOFIC schemes—the column parallel readout chain can be used for CDS of both HCG and LCG signals. In addition, since shot noise is dominant in the LCG, DRS can also be employed [39,43].

The extended DR of the LOFIC architecture, *DR*_L_, scales with increases in FWC according to the ratio of (*C*_LOFIC_ + *C*_FD_)/*C*_FD_. Therefore, the *DR*_L_ is expressed as follows:

*DR*_L_ = 20log [(*C*_LOFIC_ + *C*_FD_)/*C*_FD_]. (5)

**Figure 11:**
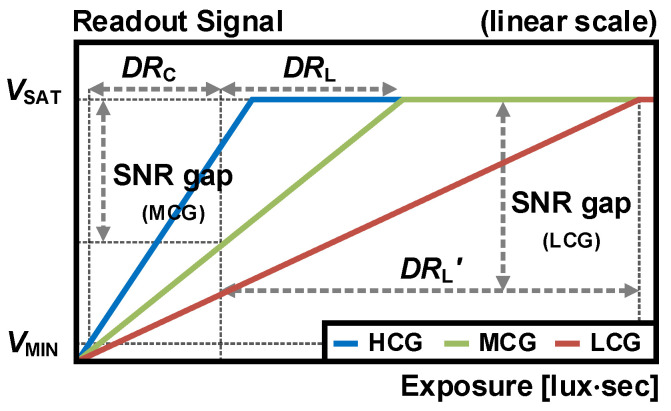
Relationship between DR and SNR gap with different CGs in LOFIC.

Figure 11 shows the correlation between the DR and SNR gap with different *C*_LOFIC_s, where the middle CG (MCG) has a smaller *C*_LOFIC_ than the LCG. The *DR*_C_ of both MCG and LCG is identical, as it is determined by the *C*_FD_, whereas the *DR*_L_ depends on the *C*_LOFIC_. For example, when *DR*_C_ = 68 dB and *C*_FD_ = 1.1 fF, a *C*_LOFIC_ of 12.6 fF (MCG) increases the *DR*_L_ by 22 dB, while a large *C*_LOFIC_ of 175 fF (LCG) increases the *DR*_L_ by 44 dB [37]. However, since the SNR gap is determined by the transitions of the MCG or LCG signals from the HCG signal, increasing the *C*_LOFIC_ results in a larger SNR gap. To ensure sufficient image quality, the DCG and LOFIC architectures need the SNR of at least 32 dB at the transition from the HCG to the LCG [37]. Therefore, an inherent trade-off between DR and SNR limits the achievable DR of LOFIC and DCG. For this reason, the triple-gain LOFIC scheme was devised with a maximum SNR drop of 4.02 dB [47].

Because the large FWC of PD is not required for the LOFIC structure, many important characteristics can be improved, such as the frame rate, operating voltage, and dark current [33,34,35,36]. Additionally, since the LOFIC needs only a single exposure, it can be non-motion artifact and flicker-free using the global shutter readout [39,40], and the multiple exposure technique can be utilized for higher DR [40]. Furthermore, combined with the DCG, high SNR can be achieved without LED flicker, and 150 dB can be achieved by imaging flicker-free objects, such as sunlight, with a short exposure [41,42]. Despite these advantages, the LOFIC architecture requires a large area per pixel due to the LG and *C*_LOFIC_. This leads to a decrease in the fill factor and makes it harder to achieve high-resolution imaging. To address this issue, a scheme where two pixels share the same FD without the SEL was devised [38]. In addition, as the process advances, over 120 dB of the DR can be achieved using lateral overflow integration trench capacitors (LOFITreCs) [44,45,46].

### 4.2. Dual Photodiode

The dual PD architecture uses different-sized PDs to improve the DR with a single exposure. With the same light intensity and integration time, the LPD can integrate more charge than the SPD, corresponding to the sensitivity ratio of two PDs. This means that the SPD behaves similarly to a short integration time of multiple exposure, as illustrated in Figure 5b and Figure 6b. Therefore, the SPD has the advantage of imaging in high-light intensity. The following covers the operation of the typical dual PD architecture with the DCG [48,49,50].

**Figure 12:**
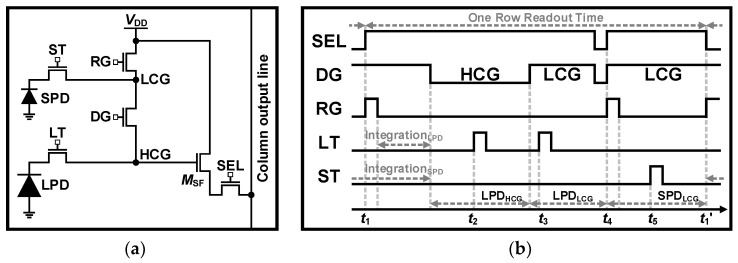
(**a**) Schematic and (**b**) timing diagram of dual PD architecture.

The dual PD schematic is depicted in Figure 12a, where the LT and ST are used for the TG, and the DG is a gate for dual gain readout. The timing diagram is illustrated in Figure 12b, where the readout of the dual PD structure is identical to the DCG structure, except that one more readout is required for the SPD signal. From *t*_1_ to *t*_4_, the LPD is read out with HCG and LCG signals, similar to the DCG scheme. Then, the RG is turned on at *t*_4_, and the noise of the SPD is sampled after the RG is turned off. At *t*_5_, the ST is switched on, and the SPD signal with LCG is read out after the ST is switched off.

The optical response between PDs increases, further extending the DR, but the SNR gap increases at the transition to the SPD signal, similar to multiple exposure [117]. Nevertheless, one of the most important advantages of the dual PD scheme over the multiple exposure technique is that it can be read out in a single exposure to mitigate motion artifacts and LED flicker. The dual PD structure can also be utilized together in a way that addresses the drawbacks of the multiple exposure technique [51]. The utilization of multiple exposure with the dual PD allows for overlapping exposures, which can address motion artifacts and LED flicker more effectively than using only multiple exposure. Moreover, the dual PD eliminates the need for extra line buffer memory to synthesize readout signals by aligning exposures. To extend the DR further, a combination of the LOFIC scheme [52,53,54,55] with organic photoconductive film (OPF) [56,118] was devised, achieving more than 120 dB DR. The primary challenge in designing submicron pixels with dual PDs is achieving high linearity in optical performance and dual gains. Also, the QE of the LPD and SPD must be precisely aligned across wavelengths within each color channel [49].

The previous and current sections describe the principles and trade-offs of design techniques and architectures that provide a linear response for WDR. The multiple exposure technique [9] achieves a DR of 100 dB by setting the *T*_long_/*T*_short_ to 119; however, it is constrained by increased power consumption and a reduced frame rate. In contrast, the dual PD scheme with DCG [52] exhibits a DR of 121 dB by employing a sensitivity ratio of 10 between the LPD and SPD, at the cost of increased process complexity. Therefore, the multiple exposure and dual PD schemes can be selected depending on the trade-off between motion artifacts/LED flicker and process cost/linearity. The DCG structure [20] enables moderate-to-high DR with a small pixel pitch of 2.8 μm while maintaining a sensitivity of 31 ke^−^/lux∙s, whereas the LOFIC method [39] achieves a large FWC of 224 ke^−^ with comparable sensitivity at the expense of pixel pitch scalability. Accordingly, the DCG and LOFIC architectures can be employed in portable and automotive applications, where the trade-off between extending the upper range of light intensity and pixel area must be balanced. Consequently, to facilitate a comparative evaluation of these technologies, the performance of typical design techniques and architectures is summarized in Table 1.

## 5. Mixing Non-Linear and Linear Responses

The subjective brightness of the human eye is logarithmic, as the theory of human visual perception suggests. This means that for intense light, the human eye perceives the change nonlinearly, like a logarithmic function. Therefore, CISs with a logarithmic response can be employed to capture images with extremely bright illumination that require over 120 dB. Therefore, they can be adopted in applications including automotive systems, robotic vision, security, scientific measurements, and the IoT, where very high DR is required. Following the previous section on CISs with a linear response that achieve WDR, this section presents CISs with a logarithmic response for use in these industries.

### 5.1. Logarithmic Response

CISs with a linear response require a relatively large pixel size, high-resolution digitizers, and in some cases, storage for WDR. CISs with a logarithmic response can be selected in applications that have these problems.

**Figure 13:**
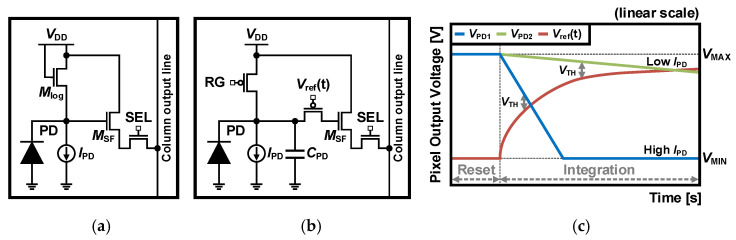
(**a**) Schematic of a logarithmic-response APS, (**b**,**c**) schematic and voltage response to photocurrent of an adjustable logarithmic-response APS [62].

The schematic of the conventional APS with logarithmic response [57,58] is depicted in Figure 13a, which is identical to the 3T APS architecture, with the RG replaced by a diode-connected scheme. In the linear response, when light enters the PD, it accumulates the photons generated. In contrast, the APS with a logarithmic response converts light into photocurrent that flows through a *M*_log_ transistor. Since this current ranges from picoampere to nanoampere, the *M*_log_ operates in weak inversion. In this region, the gate-source voltage (*V*_GS_) of the *M*_log_ depends logarithmically on the current as follows:

*V*_GS_ = (*nkT*/*q*) ∙ ln (*I*_PD_/*I*_O_) + *V*_TH_, (6)

where *n* is the ideality factor of the MOSFET, *kT*/*q* is the thermal voltage, *I*_PD_ is the photocurrent, *I*_O_ is the *I*_PD_ at the onset of weak inversion, and *V*_TH_ is the threshold voltage. The responsivity of the voltage is about 35 mV per decade of light intensity due to the body effect, resulting in a relatively small output voltage swing. As a result, CISs with a logarithmic response become dominated by the FPN due to the parameter variations caused by the fabrication process. CISs with a linear response can suppress the FPN through the CDS scheme, whereas CISs with a logarithmic response cannot, due to the discontinuous conversion of charge to voltage. For this reason, it is essential to calibrate the pixel offset for non-uniformity with the logarithmic response. The calibration of the FPN can be achieved off-chip using an external storage with an integrated co-processor [58], on-chip by sampling the photocurrent and reference current [59], or using a lateral PNP amplification pixel and in-pixel CDS [60].

CISs with a logarithmic response have the DRs of 120 dB due to the relatively small output voltage variation in response to photocurrent caused by weak inversion. However, high FPN not only constrains their performance, but they also have poor sensitivity to the dark current and a slow response due to the small photocurrent in low-light intensity. To address these issues, a scheme for a logarithmic response with reference voltage was proposed [61,62]. The schematic for [62] is shown in Figure 13b, where a PMOS gate driven by a time-dependent reference voltage is combined with the 3T APS. The readout operation first resets the RG and *V*_ret_(t) to low, making the *V*_PD_ the maximum voltage. When the RG is turned off, the photocurrent discharges the *C*_PD_ to begin integration. At the same time, the *V*_ret_(t) increases logarithmically, as illustrated in Figure 13c, and when the voltage difference between *V*_PD_ and *V*_ref_(t) reaches the *V*_TH_, the PMOS gate is turned off. In other words, the integration time is adaptively controlled by the photocurrent, resulting in a short integration time for the *V*_PD1_ (high photocurrent) and a longer integration time for the *V*_PD2_ (low photocurrent). It is also less sensitive to the FPN because of the larger output voltage swing than the logarithmic-response APS. As a result, it can achieve a WDR of up to 120 dB while operating with an effective integration time of 20 ms.

In this approach, the readout operation is conducted by the PMOS gate as the threshold comparison, i.e., it is also affected by the FPN because of the threshold-voltage variation. To mitigate this, a double-sampling technique was devised that utilizes data with rapidly increasing reference voltage when the RG is turned off [63]. Alternatively, an architecture that combines a PMOS gate driven by a time-dependent reference voltage with the 4T APS was devised to improve sensitivity under dark conditions [64], and a scheme for optimal photoresponse can also be utilized [65].

### 5.2. Linear-Logarithmic Response

**Figure 14:**
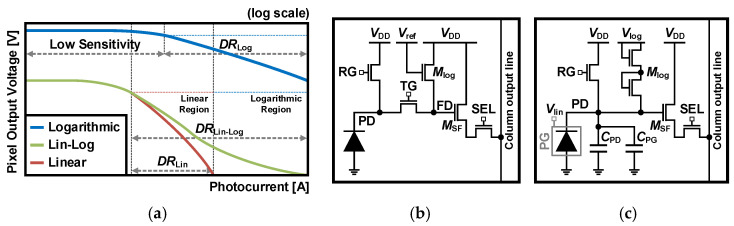
(**a**) Output voltage response to photocurrent with Lin-Log response, (**b**,**c**) schematic of adjustable Lin-Log APS [70,73].

The fundamental principle of Lin-Log response is that when a pixel is saturated with a linear response, it is replaced with the corresponding logarithmic response. This maintains the high DR of the logarithmic response while eliminating the drawbacks of high FPN and slow response. Each DR in the Lin-Log response is illustrated in Figure 14a. The linear response offers better sensitivity and SNR in dark conditions, while the logarithmic response provides smaller swing and higher DR under bright conditions, enabling a hybrid Lin-Log response to achieve WDR with the advantages of both responses.

APSs with a Lin-Log response vary depending on the scheme. One approach is to take two readouts, one linear and one logarithmic, and select data based on whether the linear signal is saturated or not [67,68]. However, this requires many transistors and aligns a linear curve to a logarithmic curve for WDR. Another approach is to combine two responses within a pixel into single data, which takes four transistors in the pixel [69]. However, this cannot independently optimize the combined data.

To mitigate these drawbacks, adjustable Lin-Log APS schemes were devised [70,71,72,73]. One method is a five-transistor APS structure, as shown in Figure 14b [70], where the *M*_log_ works in the sub-threshold region, conducting logarithmic voltage. The readout operation is performed in three phases. First, the RG and the *M*_log_ are turned on in the clear phase to remove the signal from the previous frame. Then, in the reset phase, the PD and FD are set to a voltage lower than the *V*_DD_ by the *V*_TH_. In the last exposure phase, the RG is turned off, the TG is turned on, and the *V*_ref_ changes from the *V*_DD_ to *V*_log_. During the integration time, under low illumination, the pixel functions as the 3T APS, while under high illumination, the *M*_log_ is turned on to work as the logarithmic response when the FD discharges below the *V*_TH_ than the *V*_log_. By utilizing only the *M*_log_ with the 4T APS, this scheme achieves a DR of over 140 dB with data from a single exposure. Moreover, the FPN in the logarithmic response can also be reduced, since the reset and signal include the *V*_TH_, which can be removed by the CDS. This also ensures that the swing in the linear region is tunable as the *V*_log_ changes, allowing adequate control of the linear-to-logarithmic tipping point for different conditions [71].

Another method is the 3T APS with two diode-connected transistors, where the PD surrounded by a photogate (PG), the schematic of which is shown in Figure 14c [73]. This scheme has three sensitivities, with two linear responses and a logarithmic response due to the PG and the cascoded *M*_log_. The first sensitivity, with a linear response, is the highest and provided by the *C*_PD_, similar to the 3T APS. The second sensitivity, also with a linear response, is lower and comes from the *C*_FD_ + *C*_PG_ configuration when the *V*_PD_ is less than the difference between the *V*_lin_ and the threshold voltage of the PG. The last sensitivity has a logarithmic response due to the cascoded *M*_log_ turning on when the *V*_PD_ is 2*V*_TH_ lower than the *V*_log_. This indicates that the DR can be scaled by adjusting the PG sensitivity and logarithmic response according to the bias voltages. However, while two linear sensitivities can improve the SNR, the PG causes the size of the PD to increase.

For WDR, CISs with a Lin-Log response also need to be calibrated due to the large FPN caused by the logarithmic response. However, calibration of the FPN needs additional components and image processing [58,60]. Without these costs, the FPN can also be suppressed by using an injected charge signal with the 3T APS [69]. However, this requires a longer frame time due to the settling of injected charge and additional operations. To address this, an FPN correction scheme was proposed using a two-step charge transfer operation in the 4T APS [74]. This approach takes the first readout through a relatively low TG voltage with a partial signal and reads the fully transferred signal. Then, one of the two signals is chosen for the offset FPN-free Lin-Log property. However, even though this reduces the FPN, the mismatch remains in the logarithmic response. For this reason, gain FPN correction based on characteristic deviation was proposed [75]. In addition, there are ways to combine the LOFIC with logarithmic response to achieve high SNR and low power consumption [76,77], and the DCG can be combined with logarithmic response to achieve more than 140 dB [78].

In this section, the principles and trade-offs of design techniques and architectures with a non-linear response for WDR are discussed. A logarithmic response of approximately 200 mV/decade [60,63] enables WDR of 120 dB but sacrifices signal linearity. In contrast, a Lin-Log response with linear sensitivity of about 700 mV/lux∙s [68,70] achieves WDR of 140 dB but requires calibration between the linear and logarithmic data. Consequently, these approaches are suitable for applications requiring high DR under bright conditions, where precise linearity is less critical, such as surveillance or automotive vision systems. To provide a quantitative analysis of these schemes, Table 2 summarizes the performance of non-linear responses.

## 6. Extending Dynamic Range Under Low Illumination

In previous sections, the focus was on how to increase the DR. However, industries such as life sciences, drones, security, medical, and IoT require the ability to capture clear images in dark environments under sub-lux conditions [119,120]. In low-light intensity, the SNR of CISs is restricted by the FPN, reset noise, and RN. The FPN and reset noise can easily be decreased by the CDS scheme [79,80,81]. The RN can be reduced to the sub-electron level by increasing the gain in the input stage of the readout chain and further mitigated through the CMS.

### 6.1. Increasing Conversion Gain and Readout Gain

One of the solutions for lowering the RN is to increase the CG. This can be achieved by employing the self-aligned source/drain and shallow trench isolation (STI) [82] or full-depth deep-trench isolation and locally lowered stack [83]. In addition, a pump-gate jot with a tapered reset gate can be utilized to extend the CG [84]. These approaches focus on minimizing the overlap capacitance of the FD to achieve high CG, with detailed capacitance analysis provided in [85]. Another method is to reset without the RG using the implanted n^+^ layer to eliminate the influence of the RG [86]. However, this requires a very high voltage of about 25 V to reset. For this reason, a bootstrapping reset method can be used to eliminate the need for high supply voltages for the FD reset [87].

**Figure 15:**
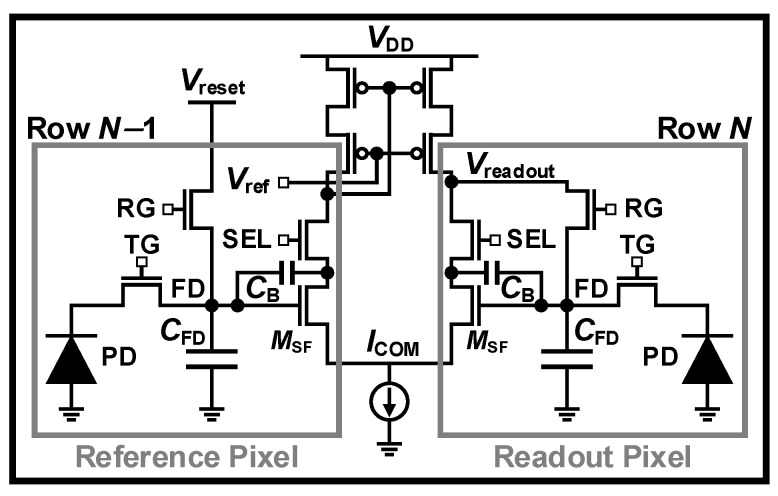
Reference-shared in-pixel differential amplifier (RSDA) [91].

The RN can also be reduced by increasing the readout gain, which is implemented by adding a high-gain stage in the column amplifier to improve performance under low-light conditions. The high-gain value of the amplifier should be optimized to balance the DR and SNR when switching between high- and low-gain modes [88,89]. For example, at a high gain of ×30, an FWC of 1300 e^−^ with an RN of 0.909 e^−^_rms_ is achieved [88]. At a low gain of ×1, the FWC increases to 37,000 e^−^, while the RN rises to 7.7 e^−^_rms_. Consequently, the DR is enhanced by about 29 dB, whereas the SNR decreases by approximately 18 dB at the transition from the high-gain to the low-gain data. However, this scheme is difficult to utilize in mobile applications, requiring small pixel pitch and low power consumption for high resolution. To address these, the SF of two rows can be utilized for use as a differential CS stage by vertically sharing two pixels [90,91]. This can operate the low and high gains with the SF and differential CS, respectively, resulting in high sensitivity without the addition of transistors. Thus, this scheme can suppress input-referred noise in dark conditions and achieve high gain by reducing the gain loss due to the body effect of the SF. However, the input of the in-pixel amplifier is doubled, which limits noise reduction. To mitigate this issue, it was proposed to connect the PMOS cascode current mirror and *I*_COM_ to the common line of each row, as shown in Figure 15. In this case, the reference pixels are parallel, resulting in a large *W*/*L* size, which reduces the noise on the differential pair to a negligible level. The CG is determined by *q*/((*C*_FD_ + *C*_B_)/*A*_v_ + *C*_B_), where *A*_v_ is the open-loop gain of the in-pixel amplifier, and *C*_B_ is the feedback capacitance when the signal is amplified with the CS. This means that since the *C*_B_ is relatively smaller than the *C*_FD_, the CG is extremely high, providing high sensitivity.

### 6.2. Correlated Multiple Sampling

CMS involves sampling the reset and signal voltages multiple times and differentiating the averages to reduce the RN, i.e., it averages the data from multiple CDS operations. The FPN and reset noise can be eliminated by CDS, but flicker noise cannot be completely removed by CDS. CMS not only decreases the RN, FPN, and thermal noise but also effectively reduces 1/*f* and random telegraph signal noises [92,93]. Furthermore, the use of a buried channel source follower can mitigate low-frequency noise and achieve a high-output swing voltage [121]. Despite these, CMS requires additional components in the readout chain, which requires additional area and power consumption. To resolve these, a pseudo-multiple sampling technique was devised [94]. It operates by replacing a conventional analog-to-digital converter (ADC) with a low-resolution ADC and sampling the signal multiple times. It can also be utilized by simply changing the slope of the ramp generator within the single-slope ADC. However, as the number of samples increases, the noise reduction is limited when the quantization noise becomes higher than the input noise standard deviation. Moreover, a variable-resolution folding-integration/cyclic ADC [95] and a digital CMS with the buried SF and high-gain column amplifier [96] were also devised to reduce the RN.

**Figure 16:**
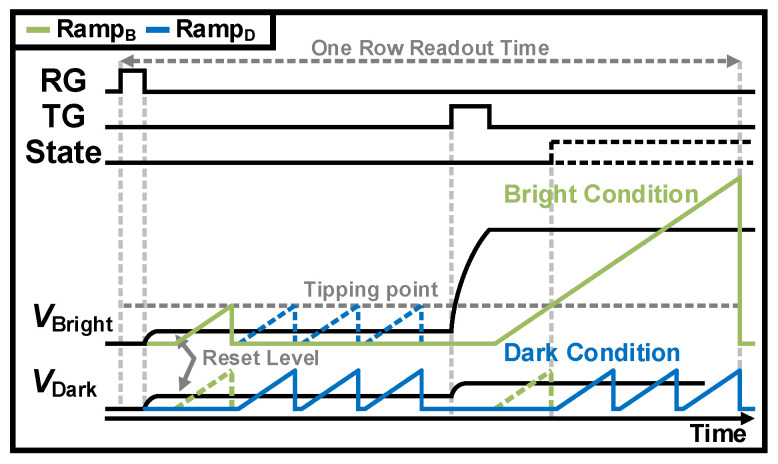
Timing diagram of conditional CMS for *M* = 4.

Conventional CMS that employs a single-slope ADC suffers from frame rate limitation due to multi-sampling. This is why a conditional CMS scheme was proposed [97,98]. The operation of conditional CMS makes multiple samplings using a small-range voltage ramp only in dark conditions, because the RN is critical in low-light intensity. Conversely, under bright conditions, a full-range voltage ramp is applied because the shot noise is dominant under high-light intensity. The timing diagram of conditional CMS is illustrated in Figure 16. After resetting, the reset level is read out once with Ramp_B_, followed by *M*–1 times with Ramp_D_, i.e., the reset signal is sampled a total of *M* times. Then, when the signal is transferred for the readout, the state is changed to high (bright condition) or low (dark condition) depending on the light intensity. This means that the state decides whether the signal is sampled once or *M* times. In further research, a digital CMS technique to sample signals into digital code [99] and an interleaved pixel SF scheme for high resolution and high frame rates [100] were also devised to reduce the RN.

As described in Section 6, increasing the CG or analog front-end gain improves sensitivity in low-light intensity but increases process complexity or power consumption. The CMS technique reduces the RN and can be readily combined with other CDS-based architectures, enabling WDR at the cost of a reduced frame rate and increased power consumption. Therefore, these schemes can be employed for industries such as medical imaging and night-vision systems, which require capturing dark scenes.

## 7. Conclusions

Advances in CMOS processes have led to multidisciplinary studies on the techniques and architectures of CIS to achieve WDR. This derives from the requirement to capture clear images in both bright and dark conditions for various industries, including automotive systems, the medical industry, machine vision, surveillance, and the IoT. Accordingly, this paper presents various mechanisms and discusses the interrelated trade-offs of each scheme. As summarized in Table 3, advanced techniques and architectures for WDR exhibit distinct performance characteristics, indicating their suitability for cutting-edge industries. Under bright conditions, WDR can be implemented by combining the DCG or LOFIC architecture with multiple exposure or dual PD. However, the need for additional exposures, readouts, or hardware can limit the frame rate and increase power consumption. Moreover, capturing images under extremely dark conditions below 0.01 lux, such as moonless environments, requires continued developments in pixel and readout circuits to further suppress noise. Consequently, continued technological advancements are required to efficiently realize WDR across the full range of illumination conditions. Specifically, an integrated research approach combining pixel architectures, readout techniques, and image signal processing algorithms can reduce low-light noise and increase high-light optical capacity, thereby improving the DR.

In conclusion, the comprehensive study of various schemes for WDR in active-pixel-type CISs provides a foundation for both industrial development and future research. Beyond typical CISs, QISs are primarily targeted at ultra-low-light imaging by detecting photoelectrons at the single-photon level, providing high sensitivity and extremely low noise. However, this scheme remains limited by FWC constraints and increased system complexity. Event-based and neuromorphic vision sensors employ asynchronous readout of intensity changes, enabling high temporal resolution and low latency at the cost of additional hardware. Although these cutting-edge sensors are not the primary focus of this paper, the design principles and trade-offs discussed for WDR provide insightful perspectives on their complementary contributions to future imaging systems.

## Figures and Tables

**Table 1:** Performance comparison of design techniques and architectures with linear response.

Design Techniques and Architectures	Pixel Size [μm^2^]	FWC [ke^−^]	Sensitivity [ke^−^/lux∙s]	DR [dB]	SNR [dB] @ Transition
Multiple Exposure [9]	5.6 × 5.6	18.5	4.1	100	14 *
DCG [20]	2.8 × 2.8	50	31	94	29 *
LOFIC [39]	3.875 × 3.875	224	36.2	88.3 **	32.3
Dual PD with DCG [52]	3 × 3	78.5	36	121	20

* Estimated from SNR curve. ** Global shutter.

**Table 2:** Performance comparison of design techniques and architectures with non-linear response.

Design Techniques and Architectures	Pixel Size [μm^2^]	Linear Sensitivity [mV/lux∙s]	Logarithmic Response [mV/decade]	Logarithmic FPN [%]	DR [dB]
Logarithmic [60]	10 × 10	-	186	8.54	120
Adjustable Logarithmic [63]	4.5 × 4.5	-	250–350	<3	>120
Lin-Log [68]	5.6 × 5.6	726	77	2	143
Adjustable Lin-Log [70]	6 × 6	651	55	1.96	144

**Table 3:** Performance comparison of advanced techniques and architectures for WDR.

Design Techniques and Architectures	Process	Pixel Size [μm^2^]	Pixel Array	DR [dB]	Frame Rate [fps]	Power Consumption [mW]
Coded Exposure [109]	65-nm	2.7 × 2.7	4224 × 4224	110	1000	7400
Pixelwise DCG [30]	350-nm	7.2 × 7.2	512 × 320	90.5	60	97.6
Dual Exposure + DCG [27]	55-nm	2.9 × 2.9	1920 × 1080	120	30	150
DCG + Logarithmic [78]	180-nm	10 × 10	512 × 512	140	>30	N/A
LOFIC + Dual PD [55]	65-nm	2.1 × 2.1	3840 × 2160	140	30	N/A
RSDA + CMS [91]	90-nm	1.45 × 1.45	3840 × 2160	78.5	35	550

## Data Availability

The data used in this paper can be obtained by contacting the first author.
